# A Novel *ABCC8* Variant in an Adult with Hyperinsulinemic Hypoglycemia Suggestive of Congenital Hyperinsulinism: A Case Report

**DOI:** 10.3390/reports9030305

**Published:** 2026-09-11

**Authors:** Hadas Rabani, Ilana Rosenblat, Khaled Osman, Adel Shalata, Leonard Saiegh

**Affiliations:** 1 Endocrinology Unit, Bnai Zion Medical Center, 47 Eliyahu Golomb Boulevard, Haifa 3339419, Israel; 2 The Simon Winter Institute for Human Genetics, Bnai Zion Medical Center, 47 Eliyahu Golomb Boulevard, Haifa 3339419, Israel; 3 The Ruth and Bruce Rappaport Faculty of Medicine, Technion-Israel Institute of Technology, Haifa 3200003, Israel

**Keywords:** congenital hyperinsulinism, hypoglycemia, SUR1

## Abstract

**Background and Clinical Significance**: Congenital hyperinsulinism (CHI) is a disorder involving excessive insulin secretion due to mutations in the insulin regulation pathways. Patients are typically diagnosed during infancy following recurrent hypoglycemic events, which may lead to irreversible neurologic damage if left untreated. Adult presentation and diagnosis are rare. **Case Presentation**: Here, we report the unusual case of a 41-year-old patient who presented with recurrent episodes of hyperinsulinemic hypoglycemia. An extensive diagnostic evaluation yielded no evidence of an acquired cause. Genetic testing revealed a heterozygous novel c.4078G>C substitution in the *ABCC8* gene, which plays a role in the insulin secretion pathway. This variant may have been the cause for CHI going unrecognized during the patient’s childhood and could have also contributed to his uncontrolled convulsive disorder. Treatment with diazoxide and octreotide led to significant clinical improvement, with no severe hypoglycemic events during the final week of hospitalization. **Conclusions:** This case emphasizes the importance of considering congenital hyperinsulinism in the differential diagnosis of adult patients evaluated for hyperinsulinemic hypoglycemia, especially when standard workup fails to identify an underlying cause.

## 1. Introduction and Clinical Significance

Hyperinsulinemic hypoglycemia in non-diabetic adults is a clinically significant condition associated with neurologic complications and impaired quality of life [1,2]. The main etiologies include medications, pancreatic insulinoma, bariatric surgery, adrenal insufficiency, insulin autoantibodies, and non-insulinoma pancreatogenous hypoglycemia syndrome (NIPHS). Once these causes are excluded, less common etiologies should be considered [1,2].

Congenital hyperinsulinism (CHI) is a disorder involving excessive insulin secretion causing hypoglycemic episodes. The majority of patients are diagnosed at the neonatal period due to recurring hypoglycemia episodes. Several mutations in key genes involved in insulin secretion and regulatory pathways have been implicated in CHI, most notably those affecting the pancreatic β-cell ATP-sensitive potassium (K-ATP) channel subunits. Different genetic mutations are associated with varying levels of clinical severity, responsiveness to diazoxide therapy, and pancreatic histological patterns [3,4].

We describe the case of a 41-year-old patient who presented with recurrent episodes of hypoglycemia associated with hyperinsulinemia, without any identifiable acquired cause despite extensive evaluation. Genetic testing revealed a novel variant in the *ABCC8* gene, which encodes the sulphonylurea receptor 1 (SUR1) subunit of the K-ATP channel and may underlie the patient’s hypoglycemic disorder. To our knowledge, this variant has not been previously described in the literature.

## 2. Case Presentation

A 41-year-old man was admitted to our hospital following a motor vehicle accident, sustaining multiple injuries, including pelvic and lumbar vertebrae fractures. At admission, his serum glucose concentration was 38 mg/dL, with negative urinary ketones. During hospitalization, serial glucose monitoring revealed recurrent episodes of hypoglycemia, although the patient remained asymptomatic. He had a body mass index (BMI) of 21 kg/m^2^, reported normal dietary intake, and denied any recent weight changes or new medications. Apart from the trauma-related injuries, physical examination was unremarkable. Laboratory investigations showed normal renal and hepatic function, as well as electrolyte levels within reference ranges.

Additional laboratory tests conducted during a hypoglycemic episode (serum glucose concentration of 45 mg/dL) revealed a non-suppressed C-peptide of 0.97 ng/mL (0.32 nmol/L), consistent with the diagnosis of hyperinsulinemic hypoglycemia. Insulin levels were inappropriately detectable (2.2 µIU/mL) [1,3]. Urinary semi-quantitative ketone levels were low (15 mg/dL).

The patient’s medical history was notable for epilepsy, characterized by frequent and poorly controlled seizures, diagnosed at one year of age. He has been treated with valproic acid and levetiracetam. Additionally, he had a diagnosis of mild intellectual disability; although living independently, he received support from his siblings and social services. Additional comorbidities included active tobacco use, psoriasis, and dyslipidemia.

During neurological follow-up for his seizure disorder, repeated electroencephalograms (EEGs) showed no evidence of epileptiform activity. At 38 years of age, incidental hypoglycemia was identified when reviewing previous routine blood tests, prompting referral for endocrine evaluation. The patient subsequently underwent a supervised fasting test, during which asymptomatic hypoglycemia was documented after six hours, with a serum glucose concentration of 36 mg/dL. At the time of hypoglycemia, the C-peptide concentration was 0.43 nmol/L, indicative of hyperinsulinemic hypoglycemia, and the insulin concentration was again borderline, 2.3 µIU/mL [1,3]. A 1 µg cosyntropin stimulation test demonstrated a normal cortisol response, effectively ruling out adrenal insufficiency. In order to evaluate for a possible insulinoma, a pancreatic protocol abdominal CT scan was performed, revealing two small hypodense foci in the pancreas without pathological contrast enhancement. An endoscopic ultrasound (EUS) was attempted but was technically unsuccessful. The patient was advised to undergo pancreatic magnetic resonance imaging (MRI); however, he was lost to follow-up.

Two years later, the patient was admitted to another hospital following a convulsive episode that resulted in multiple traumatic injuries. During this admission, recurrent episodes of hypoglycemia were again documented. At the time of hypoglycemia (serum glucose concentration of 37 mg/dL), laboratory evaluation revealed a C-peptide concentration of 0.46 nmol/L and an insulin concentration of 6.22 µIU/mL, both consistent with the diagnosis of hyperinsulinemic hypoglycemia [1]. A repeat pancreatic protocol abdominal CT scan was performed and was interpreted as normal. The patient was discharged on diazoxide therapy at a dose of 100 mg three times daily, and was advised to undergo further evaluation with pancreatic MRI and a Gallium-68-DOTATATE PET-CT scan. However, he was again lost to follow-up.

The patient was admitted to our hospital one year later, as described above, and underwent further evaluation to clarify the etiology of his recurrent hypoglycemia. A urine screen for sulfonylureas performed during a hypoglycemic episode was negative, as was testing for insulin autoantibodies. EUS was performed and did not demonstrate any suspicious pancreatic lesion. In addition, a thorough review of the patient’s active medications during hospitalization confirmed that none were known to be associated with hypoglycemia [1,2].

During the first week of hospitalization, six hypoglycemic events were documented (serum glucose < 70 mg/dL), three of which were classified as severe (serum glucose < 55 mg/dL) [1]. These events occurred at various times throughout the day, including during the morning fast, but were most frequently observed in the afternoon.

Treatment with diazoxide was initiated and gradually titrated to a high dose of 150 mg three times daily (approximately 8 mg/kg/day). This regimen resulted in partial improvement, with continued hypoglycemic episodes occurring 3–6 times per week, though these were less severe. Due to the suboptimal response, octreotide was initiated at a dose of 100 mcg three times daily, leading to further improvement in glycemic control. During the final week of hospitalization, only one mild hypoglycemic episode occurred.

In view of the overall findings, a previously undiagnosed CHI was considered. Retrospective review revealed documented hypoglycemia as early as 19 years of age, with a serum glucose concentration of 56 mg/dL recorded during a hospital visit following a convulsive episode. Unfortunately, no earlier laboratory data or perinatal records were available for review. The patient had no known family history of hypoglycemic disorders. There was no history of parental consanguinity or relevant familial disease, and he has two healthy siblings.

Genetic testing was performed using targeted next-generation sequencing (NGS) on a hypoglycemia and hyperinsulinism associated gene panel, using the IDT xGen Exome Research Panel V2 Target Regions combined with an mtDNA panel. Library preparation was conducted using the Nextera DNA Flex Pre-Enrichment Library prep, followed by paired-end sequencing on an Illumina NovaSeq 6000 platform (Illumina, San Diego, CA, USA).

The test revealed a heterozygous missense variant in the *ABCC8* gene, c.4078G>C replacement, which results in a valine-to-leucine substitution at position 1360 (p.Val1360Leu). The entire *ABCC8* coding region was covered at a minimum sequencing depth of 10×. The *ABCC8* gene encodes the SUR1 subunit of the K-ATP channel in pancreatic β-cells, and inactivating mutations in this gene are a well-established cause for CHI. To our knowledge, this variant has not been previously described in the literature. It is located within the gene’s mutational hot domain and is expected to have a deleterious effect on the gene product. Taken together, these findings suggest the novel *ABCC8* variant as a likely candidate for causing the patient’s hyperinsulinemic hypoglycemia.

At discharge, treatment with monthly long-acting lanreotide was recommended in combination with high-dose diazoxide. The patient continued follow-up at our clinic for seven months before being lost to follow-up. During this period, he remained on diazoxide and received lanreotide intermittently. Although he did not perform home glucose monitoring, he reported no convulsive episodes throughout the follow-up period.

## 3. Discussion

In this report, we present the case of an adult patient who was evaluated for hyperinsulinemic hypoglycemia. Hypoglycemia was first recognized and evaluated at the age of 38, and supervised fasting test suggested hyperinsulinemia. Pancreatic protocol abdominal CT revealed two pancreatic nodules, yet EUS was technically unsuccessful. Two years later, he was hospitalized due to a convulsive episode, and hypoglycemic events with high insulin and C-peptide were documented. A repeat CT scan was normal, and although further imaging was recommended, the patient was lost to follow-up. After one year, he was admitted to our hospital, and recurrent hypoglycemia was again documented. EUS showed no suspicious pancreatic lesions, and further evaluation, including a cosyntropin stimulation test, insulin antibody assay, and urine sulfonylurea screen, were negative.

As extensive diagnostic workup yielded no evidence of an acquired etiology, genetic testing was pursued. A novel heterozygous c.4078G>C variant in the *ABCC8* gene was identified, located within a known mutational hotspot. The findings are consistent with the possible pathogenic role of this variant, underlying the patient’s hyperinsulinemic hypoglycemia.

This case is notable for the exceptionally late diagnosis of CHI at the age of 41. Most cases of CHI present in infancy or early childhood, with the majority diagnosed during the neonatal period due to severe hypoglycemia [3]. Other cases of CHI diagnosis in adulthood have been reported only rarely in the literature [5,6,7,8]. Early diagnosis and treatment of hypoglycemic disorders are critical, as recurrent hypoglycemia can lead to central nervous system injury, including seizure disorders, neurodevelopmental delays, and irreversible cognitive impairment [3,4]. In our case, the patient was diagnosed with a convulsive disorder at the age of one year, described as difficult to control despite multiple antiepileptic therapies. Review of his past medical history revealed low glucose levels documented in some emergency room visits for convulsive episodes or traumatic accidents; however, childhood medical records were unavailable. It is possible that this early-onset seizure disorder resulted from unrecognized hypoglycemic episodes. If so, prolonged unnoticed hypoglycemia may also account for the patient’s intellectual disability.

The diagnosis of hyperinsulinemic hypoglycemia requires documentation of non-suppressed insulin and C-peptide levels during a hypoglycemic episode [1]. In our case, C-peptide levels were consistently above the diagnostic threshold of 0.2 nmol/L, while insulin exceeded the diagnostic threshold of 3 µIU/mL in only one critical sample, yet was above 2 µIU/mL in the other two. Given insulin’s short half-life, low insulin levels during hypoglycemia have been reported, and a lower threshold of 2 µIU/mL has been proposed [3,4]. Unfortunately, serum proinsulin, free fatty acids, and β-hydroxybutyrate measurements were not available. Due to the patient’s poorly controlled convulsive disorder, hypoglycemia was promptly treated and a glucagon stimulation test was therefore not performed.

A genetic etiology is identified in approximately 45–80% of CHI cases, depending on the diagnostic methods employed [3]. To date, mutations in more than 15 genes have been associated with CHI, displaying a broad spectrum of clinical phenotypes. They are typically classified based on severity, responsiveness to diazoxide treatment and pancreatic histopathology [3,4]. Pathogenic variants in the *ABCC8* and *KCNJ11* genes, encoding the two subunits of the K-ATP channels in pancreatic β-cells, represent the most common genetic cause of CHI and account for approximately 50% of cases [9].

The *ABCC8* gene, located on chromosome 11p, encodes the SUR1 regulatory subunit of the K-ATP channel. Under physiological conditions, hyperglycemia leads to increased intracellular ATP production, raising the ATP/ADP ratio. SUR1 senses this change and mediates closure of the K-ATP channels, resulting in membrane depolarization and subsequent insulin secretion [9,10]. Loss-of-function mutations in *ABCC8* disrupt this regulatory pathway by two principal mechanisms. One mechanism involves impaired SUR1 synthesis, maturation, or trafficking, leading to reduced K-ATP channel surface expression. The second results from disruption in MgADP-dependent channel activation, causing an inappropriate closure of the K-ATP channel despite low glucose levels and consequently unregulated insulin secretion. To date, over 400 *ABCC8* variants have been associated with CHI, displaying a wide spectrum of clinical severity [9,10].

**Figure 1:**
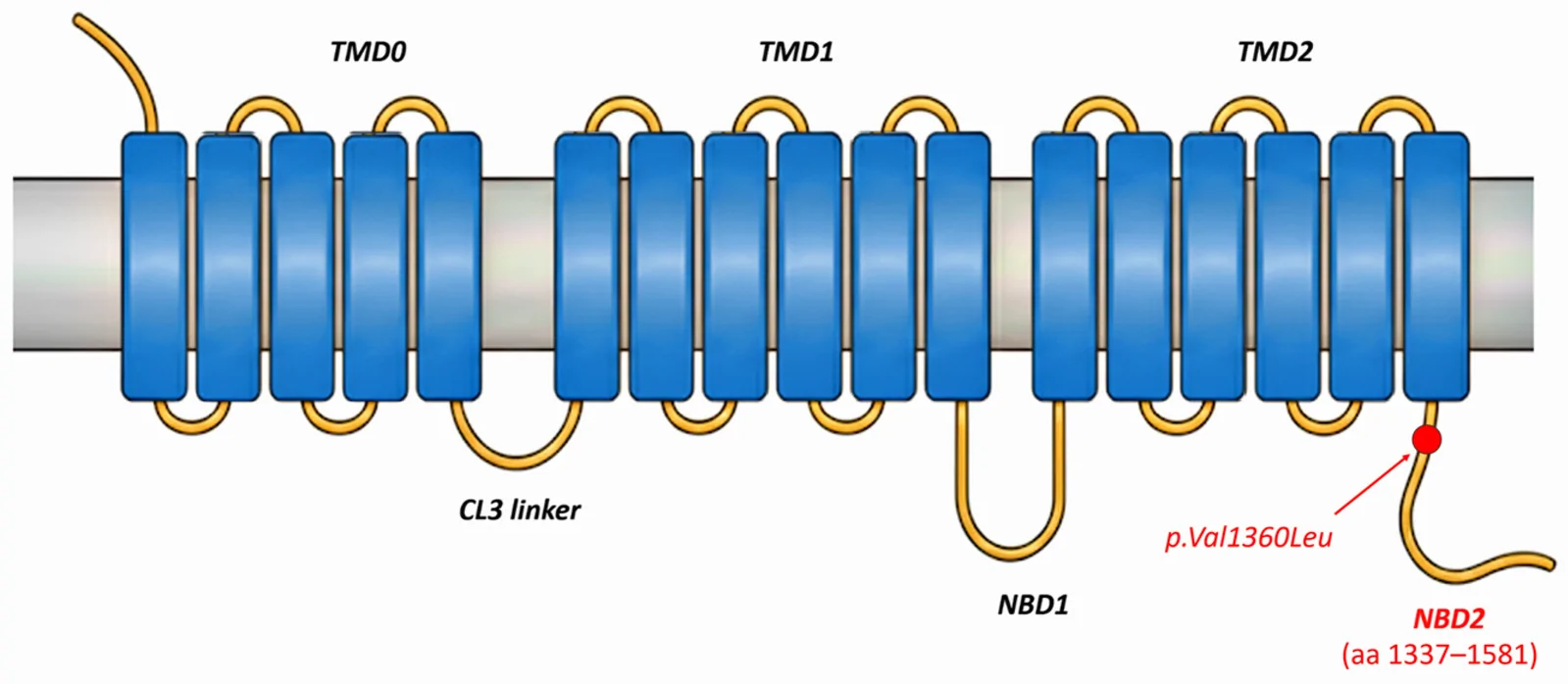
Schematic of the *ABCC8* gene product. Each SUR1 subunit includes three transmembrane domains (TMDs), two nucleotide-binding domains (NBDs), and cytosolic loop 3 (CL3) linker [14]. The p.Val1360Leu replacement is located in the NBD2 domain (indicated by a red dot and arrow). aa = amino acids.

Our patient was found to carry a heterozygous *ABCC8* variant, p.Val1360Leu—a novel variant not previously described in the literature [11]. This variant is absent from reference population datasets in gnomAD (v4.1.1) [12]. Multiple computational prediction tools indicated a deleterious effect of the variant on protein function, including a REVEL score of 0.86 and an AlphaMissense score of 0.957. The variant is located within the Nucleotide Binding Domain 2 (NBD2), the principal binding site for ADP on the SUR1 receptor and a well-recognized hotspot for CHI-associated mutations, enriched for pathogenic *ABCC8* variants (see Figure 1) [10,13]. Furthermore, the p.Val1360Met variant, a different missense change at the same residue, was previously reported to be pathogenic in a patient with CHI [14].

Medical genetics experts who evaluated this case classified the variant as likely pathogenic according to the ACMG/AMP-based scoring system. The classification was supported by the following evidence criteria: PM1_Moderate (2 points), PM2_Supporting (1 point), PM5_Supporting (1 point), PP3_Moderate (2 points), and PP4_Supporting (1 point). The resulting overall score exceeded the predefined threshold of 6 for classification as likely pathogenic. [15].

The first-line treatment for CHI is diazoxide, an oral nondiuretic benzothiadiazine that suppresses insulin secretion by opening the beta-cell K-ATP channels. Fluid retention is a common adverse effect, and may limit dose titration or require the addition of a diuretic. In some patients, diazoxide requirements decrease over time, allowing dose reduction without compromising metabolic control [3,16].

While homozygous mutations in *ABCC8* are typically associated with diazoxide-unresponsive CHI, milder phenotypes, often caused by autosomal dominant mutations, tend to retain partial K-ATP channel function and are usually diazoxide-responsive [9,10]. Pinney et al. described sixteen familial cases of CHI caused by autosomal dominant mutations in K-ATP channel subunits [10]. All mutations involved single-amino-acid substitutions, and most patients responded well to diazoxide therapy [10]. One case described a 17-month-old infant who was evaluated for refractory seizure disorder and recurrent hypoglycemic episodes and found to carry a heterozygous E1507K mutation in the *ABCC8* gene. Subsequent family screening revealed eight additional mutation carriers with variable degrees of hypoglycemia. Notably, the patient’s grandfather and his twin brother were diagnosed with CHI at the age of 65, both with a prior history of seizure disorders but no recorded history of hypoglycemic episodes [10]. In our case, treatment with diazoxide resulted in partial improvement, consistent with the assumption that the patient’s heterozygous *ABCC8* mutation leads to partial loss of channel function.

The second-line treatment for CHI is somatostatin analogs. These agents activate SSTR2 and SSTR5 on pancreatic β-cells, thereby inhibiting insulin secretion through the suppression of the cAMP signaling pathway and reducing insulin gene promoter activity. Their use is limited by tachyphylaxis and gastrointestinal adverse effects. In the pediatric population, treatment may also be associated with necrotizing enterocolitis and impaired growth [3,16]. In our case, octreotide treatment was administered during hospitalization as multiple daily injections, resulting in significant improvement in metabolic control. The patient was subsequently discharged on lanreotide, a long-acting somatostatin analogue, in combination with diazoxide. Other agents are currently under investigation for CHI treatment, including long-acting glucagon formulations, insulin receptor antibodies, PI3K inhibitors and GLP-1 receptor antagonists [16].

Most cases involving *ABCC8* mutations are associated with diffuse pancreatic beta-cell dysfunction, for which total pancreatectomy may be considered as a last resort treatment in refractory cases. Focal lesions have been reported in the context of paternally inherited heterozygous *ABCC8* mutation combined with uniparental disomy of chromosome 11p15, and for such cases, partial pancreatectomy is the recommended treatment [3,4]. In the present case, imaging modalities—including pancreatic protocol CT and EUS—did not identify a focal lesion. Functional imaging with 18-fluoro-dopa PET is also recommended for the detection of focal lesions, but was not performed in this case [3].

This case study has several limitations. In vitro studies are needed to further characterize the pathogenicity of the identified *ABCC8* variant and confirm its role as a loss-of-function mutation. Moreover, the genetic analysis in our case was limited to a targeted panel; therefore, additional relevant genetic alterations cannot be excluded. Additionally, due to limited cooperation, the genetic testing of other family members could not be performed, limiting our ability to assess the mutation’s penetrance. It should be noted that even though the pancreatic protocol abdominal CT scan and EUS were negative, occult insulinoma cannot be definitively excluded, particularly since the patient did not comply with our recommendation to undergo abdominal MRI and functional imaging. Additionally, long-term outcomes are limited since the patient was lost to follow-up.

## 4. Conclusions

This case report highlights the importance of considering CHI in the differential diagnosis of adult patients presenting with hyperinsulinemic hypoglycemia, particularly when standard evaluations fail to identify an underlying cause. Although CHI is primarily diagnosed in infancy, milder phenotypes may remain unrecognized or be misdiagnosed as epilepsy or other neurological disorders [3,4]. Establishing the diagnosis not only enables tailored therapeutic interventions but also provides valuable genetic insights for family screening and reproductive counseling. Furthermore, it helps prevent unnecessary and potentially invasive investigations for alternative etiologies. As genetic testing becomes increasingly accessible and cost-effective, its incorporation into the diagnostic algorithm for unexplained hypoglycemia is both practical and clinically beneficial.

## Data Availability

The original data presented in this study are available on reasonable request from the corresponding author. The data are not publicly available due to privacy concerns.

## Abbreviations

The following abbreviations are used in this manuscript:

BMI	Body mass index
CHI	Congenital hyperinsulinism
EEG	Electroencephalogram
EUS	Endoscopic ultrasound
K-ATP	ATP-sensitive potassium channel
MRI	Magnetic resonance imaging
NBD2	Nucleotide binding domain 2
NGS	Next-generation sequencing
NIPHS	Non-insulinoma pancreatogenous hypoglycemia syndrome
SUR1	Sulphonylurea receptor 1
aa	Amino acid
